# Identification of the Key Functional Domains of *Bombyx mori* Nucleopolyhedrovirus IE1 Protein

**DOI:** 10.3390/ijms231810276

**Published:** 2022-09-07

**Authors:** Zhi-Gang Hu, Zhan-Qi Dong, Jiang-Hao Miao, Ke-Jie Li, Jie Wang, Peng Chen, Cheng Lu, Min-Hui Pan

**Affiliations:** 1 State Key Laboratory of Silkworm Genome Biology, Southwest University, Chongqing 400716, China; 2 Key Laboratory for Sericultural Biology and Genetic Breeding, Ministry of Agriculture, Southwest University, Chongqing 400716, China

**Keywords:** *Bombyx mori* nucleopolyhedrovirus, IE1, functional domain, nuclear localization element, transactivation

## Abstract

The immediate early protein 1 (IE1) acts as a transcriptional activator and is essential for viral gene transcription and viral DNA replication. However, the key regulatory domains of IE1 remain poorly understood. Here, we analyzed the sequence characteristics of *Bombyx mori* nucleopolyhedrovirus (BmNPV) IE1 and identified the key functional domains of BmNPV IE1 by stepwise truncation. Our results showed that BmNPV IE1 was highly similar to *Autographa californica* nucleopolyhedrovirus (AcMNPV) IE1, but was less conserved with IE1 of other baculoviruses, the C-terminus of IE1 was more conserved than the N-terminus, and BmNPV IE1 was also necessary for BmNPV proliferation. Moreover, we found that IE1_158–208_ was a major nuclear localization element, and IE1_1–157_ and IE1_539–559_ were minor nuclear localization elements, but the combination of these two minor elements was equally sufficient to fully mediate the nuclear entry of IE1. Meanwhile, IE1_1–258_, IE1_560–584_, and the association of amino acids 258 and 259 were indispensable for the transactivation activity of BmNPV IE1. These results systematically resolve the functional domains of BmNPV IE1, which contribute to the understanding of the mechanism of baculovirus infection and provide a possibility to synthesize a small molecule IE1-truncated mutant as an agonist or antagonist.

## 1. Introduction

Baculoviruses are a family of DNA viruses with circular double-stranded genomes that range in size from 80 to 180 kb and encode 90–180 genes [1]. To date, baculoviruses have been identified from over 700 host species, and more than 172 baculovirus genomes have been completely sequenced [2]. According to the host species and morphology of the virions, baculoviridae is divided into four genera, Alpha-baculovirus (lepidopteran nucleopolyhedrovirus), Beta-baculovirus (lepidopteran granulovirus), Gamma-baculovirus (hymenopteran nucleopolyhedrovirus), and Delta-baculovirus (dipteran nucleopolyhedrovirus). *Autographa californica* nucleopolyhedrovirus (AcMNPV) and *Bombyx mori* nucleopolyhedrovirus (BmNPV) are two representative species of Baculoviridae that are widely used as protein expression tools or gene delivery vectors for protein production, drug screening, and gene therapy [3,4]. As the host species of baculoviruses are mostly agricultural and forestry pests, baculoviruses have also been exploited as biological pesticides [5,6]. Therefore, it is crucial to investigate the mechanism of baculovirus infection to promote its application.

The infection process of baculovirus is divided into invasion, transcription, DNA replication, and nucleocapsid assembly and release, all of which are regulated by the cascade expression of viral genes. According to the phase of viral gene transcription, they are divided into immediate-early, delay-early, late, and very-late genes. Immediate early protein 1 (IE1), as a transcriptional activator of early genes, is employed by many baculoviruses to initiate a transcriptional cascade that starts the baculovirus replication cycle. IE1 can also activate the transcription of late genes [7]. Hence, the functional and structural characterization of IE1 is important for understanding baculovirus infection. Initially, IE1 of AcMNPV (AcIE1) was identified to contain at least two separable domains, a domain localized within the N-terminal 145 amino acids of AcIE1, which is essential for transactivation, while the other localized within the C-terminal 437 amino acids of AcIE1, which is required for DNA binding activity [8]. Subsequently, the N-terminal 1–125 amino acids and 168–222 amino acids of AcIE1 were identified as independent transcriptional activation domains, while residues 152–161 (basic domain I) are essential for AcMNPV homologous region (hr) enhancer binding and hr-dependent transactivation [9,10]. Moreover, the N-terminal 23 residues of AcIE1 are indispensable for origin-specific DNA replication and AcMNPV propagation [11]. As for the C-terminal sequence of AcIE1, a helix-loop-helix (HLH)-like domain extending from 543–568 amino acids mediates oligomerization of AcIE1 and is a requisite for DNA binding and transactivation [12,13]. As a transcriptional activator, nuclear transport of IE1 is essential for productive infection, and residues 534 to 538 (basic domain II) have been identified as a nuclear localization signal that is vital for nuclear entry and promoter activation [14]. Although many studies have been conducted on the function of IE1, these results are relatively isolated and it is still difficult to obtain a holistic understanding of the functional domains of IE1.

As a silk-producing insect, the silkworm has important economic value. However, BmNPV, as a serious pathogen, causes considerable economic losses annually. Using IE1 as a Clustered Regularly Interspaced Short Palindromic Repeats (CRISPR) target site is an effective strategy to increase BmNPV resistance in the silkworm; however, the knockout of different regions of the IE1 gene has been shown to have different effects on the inhibition of BmNPV [15]. The N-terminal 267 amino acids truncated mutant of BmNPV IE1 acts as an antagonist of IE1, which might serve as a drug for the treatment of BmNPV infection [16]. Therefore, in this study, the sequence characteristics of IE1 of BmNPV were identified, the effect of IE1 in viral proliferation was studied by constructing IE1 deletion and IE1 repair recombinant viruses, and finally, the nuclear localization elements and key transcriptional activation domains of IE1 were exhaustively explored by stepwise truncation. Our results comprehensively identify the functional domains of BmNPV IE1 and provide different insights into the functional domains of AcIE1.

## 2. Results

### 2.1. Sequence Characterization of BmNPV IE1

**Figure 1:**
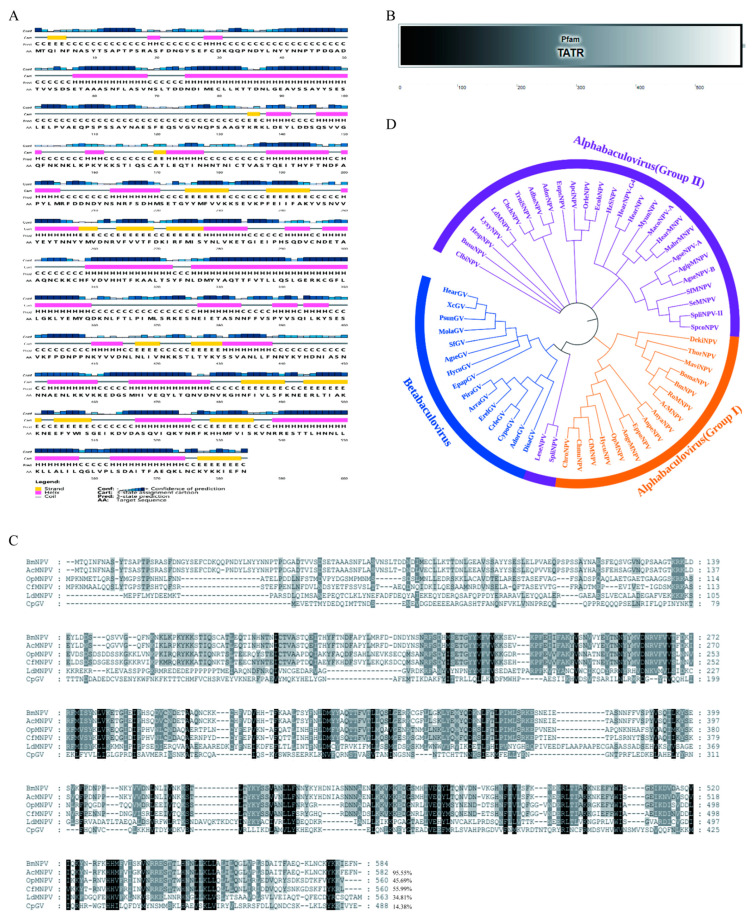
Sequence characterization of BmNPV IE1. (**A**) Secondary structure of BmNPV IE1 predicted by PSIPRED software (http://bioinf.cs.ucl.ac.uk/psipred/, accessed on 12 April 2021). The first row indicates the confidence of prediction; the second row indicates the cartoon pattern of prediction, yellow rectangle indicates strand structure, pink rectangle indicates helix structure, gray straight line indicates coil structure; the third row indicates the secondary structure prediction; the fourth row indicates the BmNPV IE1 protein sequence. (**B**) Domain of BmNPV IE1 predicted using SMART software (http://smart.embl.de/, accessed on 12 April 2021). (**C**) Homology analysis of BmNPV IE1 using ClustalX software. Highlights with different background colors represent differences in amino acid conservation. Amino acids highlighted in black indicate that the properties of these amino acids are highly consistent, and amino acids marked in gray indicate that the properties of these amino acids are similar. (**D**) Phylogenetic tree of BmNPV IE1 constructed by MEGA-X software.

BmNPV IE1 encodes 584 amino acids and has a molecular weight of 66.9 kDa. To understand the sequence characteristics of BmNPV IE1, the secondary structure of BmNPV IE1 was first predicted using PSIPRED software. The secondary structure elements of IE1 were found to be evenly distributed throughout the protein sequence (Figure 1A), which was consistent with the predicted domain of IE1 by SMART software. The 1–579 amino acids of IE1 represent the typical transactivation transcriptional regulator domain (TATR) (Figure 1B). Subsequently, sequence homology comparisons were performed between BmNPV IE1 and IE1 of AcMNPV, *Orgyia pseudotsugata* multiple nucleopolyhedrovirus (OpMNPV), *Choristoneura fumiferana* multiple nucleopolyhedrovirus (CfMNPV), *Lymantria dispar* multiple nucleopolyhedrovirus (LdMNPV) of Alpha-baculovirus, and IE1 of *Cydia pomonella* granulovirus (CpGV) of Beta-baculovirus. The results showed that the sequence similarity of IE1 varied widely among different species, ranging from 14 to 95%; the N-terminal end of IE1 was highly variable and the C-terminal end of IE1 showed a higher amino acid homology (Figure 1C). The phylogenetic tree of BmNPV IE1 was further constructed by MEGA-X software to determine the phylogenetic relationship of IE1 among different baculoviruses. As a result, the IE1 of the Alpha-baculovirus Group I in which BmNPV IE1 was located, clustered into one branch, but was distant from the IE1 of the Alpha-baculovirus Group II and Beta-baculovirus, which was consistent with the results of protein homology analysis (Figure 1D).

### 2.2. Effect of IE1 on the Proliferation of BmNPV

**Figure 2:**
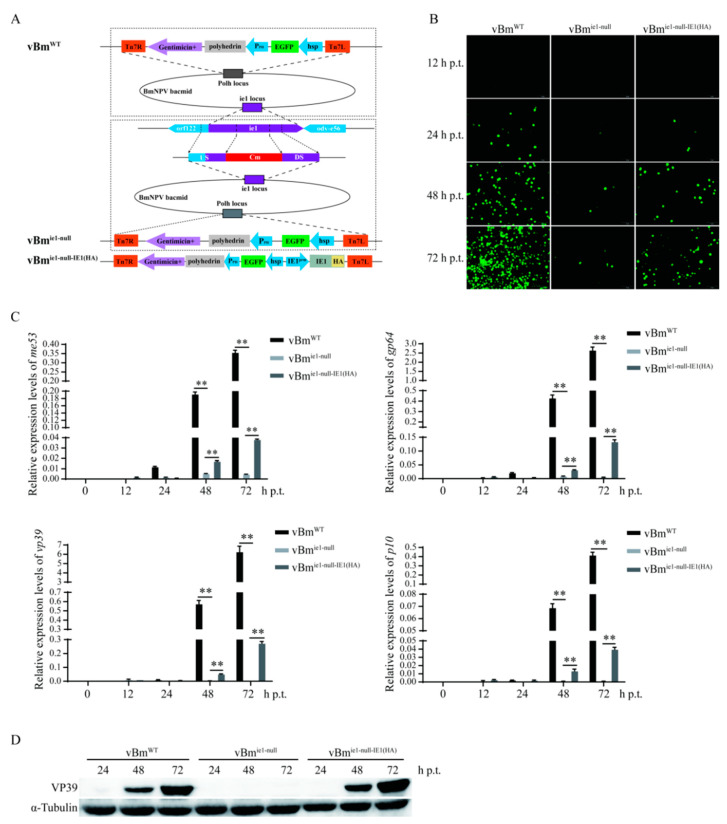
Effect of IE1 on the proliferation of BmNPV. (**A**) Schematic of the construction of wild-type BmNPV vBm^WT^, IE1 deletion virus vBm^ie1-null^, and IE1 repair virus vBm^ie1-null-IE1(HA)^ with EGFP. (**B**) Fluorescence observation of BmN-SWU1 cells. vBm^WT^, vBm^ie1-null^, and vBm^ie1-null-IE1(HA)^ bacmids were transfected into BmN-SWU1 cells. At 12, 24, 48, and 72 h p.t., EGFP-positive cells were observed by fluorescence microscopy to determine the proliferation of BmNPV. The magnification is 10 × 10. (**C**) Transcriptional levels of viral immediate-early gene *me53*, delay-early gene *gp64*, late gene *vp39*, and very-late gene *p10* in BmN-SWU1 cells transfected with vBm^WT^, vBm^ie1-null^, or vBm^ie1-null-IE1(HA)^ bacmids at 0, 12, 24, 48, and 72 h p.t. (** *p* < 0.01). (**D**) Expression level of viral protein VP39 in BmN-SWU1 cells transfected with vBm^WT^, vBm^ie1-null^, or vBm^ie1-null-IE1(HA)^ bacmids at 24, 48, and 72 h p.t.

Deletion and repair viruses are widely used for functional studies of viral genes. Here, IE1 deletion and IE1 repair recombinant viruses were constructed by homologous recombination and Bac-to-Bac technology, named vBm^ie1-null^ and vBm^ie1-null-IE1(HA)^, respectively, while BmNPV with a green fluorescent protein tag (EGFP) was named vBm^WT^ (Figure 2A). To verify whether the IE1 deletion viruses were successfully constructed, different primer pairs near the *ie-1* locus were used to amplify the target fragment. The results of agarose gel electrophoresis showed that there were differential bands at vBm^WT^ and vBm^ie1-null^, and the IE1 deletion virus was successfully constructed (Figure S1A). The IE1 repair virus was also demonstrated to be successfully constructed by immunofluorescence and Western blotting (Figure S1B). The bacmids of vBm^WT^, vBm^ie1-null^, and vBm^ie1-null-IE1(HA)^ were transfected into BmN-SWU1 cells to detect the effect of IE1 on BmNPV proliferation. Fluorescence analysis revealed that the number of EGFP-positive cells increased continuously in the vBm^WT^ group at 24 h, 48 h, and 72 h post-transfection (h p.t.), while the number of EGFP-positive cells was few and almost unchanged in the vBm^ie1-null^ group. The EGFP-positive cells in the vBm^ie1-null-IE1(HA)^ group were less than those in the vBm^WT^ group, but significantly more than those in the vBm^ie1-nul^^l^ group, and showed an increasing trend (Figure 2B). Subsequently, the effect of IE1 on BmNPV proliferation was analyzed by real-time fluorescent quantitative PCR (qRT-PCR), and the transcript levels of BmNPV immediate-early gene *me53*, delay-early gene *gp64*, late gene *vp39*, and very late gene *p10* were examined. The results showed that after 48 h and 72 h of bacmid transfection, the transcription of the viral genes in the vBm^ie1-null^ group was almost abolished, and only very low expression of *me53*, *gp64*, *vp39*, and *p10* was detected. In the vBm^ie1-null-IE1(HA)^ group, the expression levels of *me53*, *gp64*, *vp39*, and *p10* was significantly higher than those in the vBm^ie1-null^ group (Figure 2C). Furthermore, the protein levels of VP39 in the vBm^WT^, vBm^ie1-nul^^l^, and vBm^ie1-null-IE1(HA)^ groups were detected. In the vBm^ie1-null^ group, VP39 was not detected at 24, 48, and 72 h p.t., whereas in the vBm^ie1-null-IE1(HA)^ group, VP39 was detected at 48 h p.t., and the expression of VP39 increased significantly at 72 h p.t. (Figure 2D). As complete repair of IE1 deletion was not achieved using the commercial IE1 promoter, we next investigated the initiation efficiency of IE1 transcription by IE1 promoters of different lengths. Based on the prediction of promoter prediction software, three IE1 promoters of different lengths were selected, and the promoter spanning region –1 to –1987 was named IE1^prm1^, the promoter spanning region –1 to –1623 was named IE1^prm2^, and the promoter spanning region –1 to –1122 was named IE1^prm3^. The promoter spanning region –1 to –628 was named IE1^prm^, which is the commercial IE1 promoter used in the previous section (Figure S2A). These promoters were used to construct the reporter vectors psL1180-IE1^prm^-DsRed, psL1180-IE1^prm1^-DsRed, psL1180-IE1^prm2^-DsRed, and psL1180-IE1^prm3^-DsRed, respectively, to detect the promoter activity. The results of fluorescence analysis showed that the promoter activities of IE1^prm1^ and IE1^prm3^ were significantly lower than that of IE1^prm^, which was consistent with the qRT-PCR results of the *DsRed* transcript level, and the qRT-PCR results also demonstrated that the promoter activity of IE1^prm2^ was significantly higher than that of IE1^prm^ (Figure S2B). Then, the IE1 repair virus vBm^ie1-null-IE1prm2-IE1^ was constructed using IE1^prm2^. The bacmids of vBm^WT^, vBm^ie1-null^, vBm^ie1-null-IE1(HA)^, and vBm^ie1-null-IE1prm2-IE1^ were transfected into BmN-SWU1 cells, and virus proliferation was observed at 48, 72, and 96 h p.t. by fluorescence microscopy. The results showed that the number of EGFP-positive cells was significantly higher in the vBm^ie1-null-IE1prm2-IE1^ group than in the vBm^ie1-null-IE1(HA)^ group at 72 and 96 h p.t. (Figure 2C); this was consistent with the viral DNA content being significantly higher in the vBm^ie1-null-IE1prm2-IE1^ group than in the vBm^ie1-null-IE1(HA)^ group (Figure S2D). The above results indicate that the deletion of IE1 almost abolished the proliferation of BmNPV and that IE1 was essential for the BmNPV infection.

### 2.3. Identification of the Nuclear Localization Elements of BmNPV IE1

**Figure 3:**
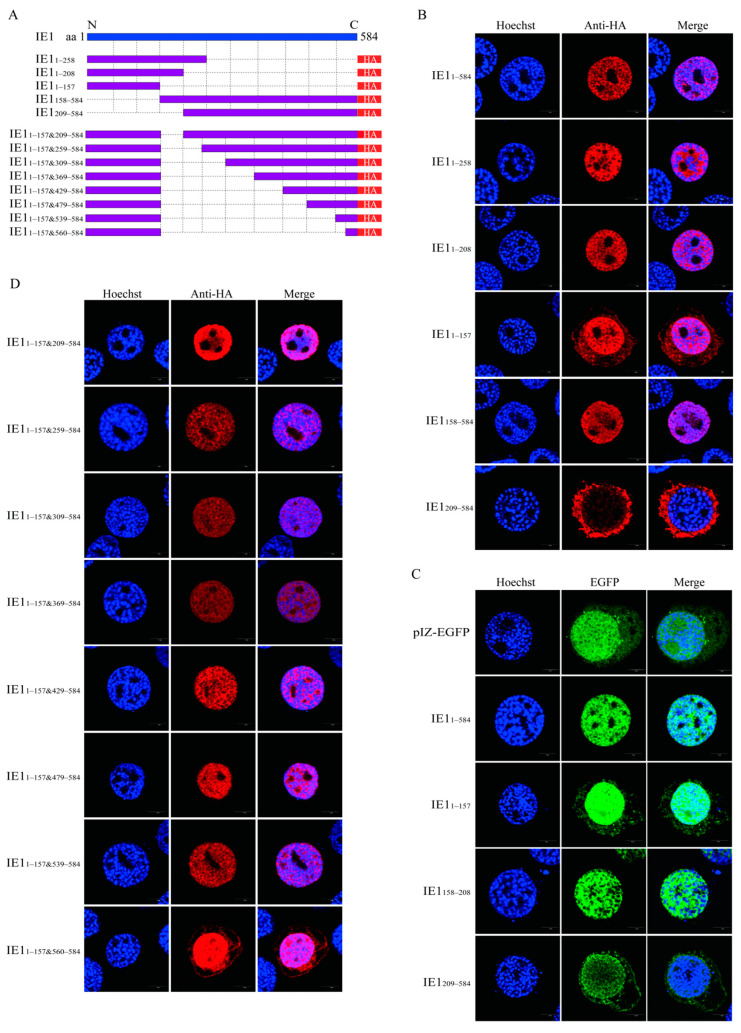
Identification of nuclear localization elements of BmNPV IE1. (**A**) Schematic of the construction of IE1 truncated mutants. (**B**) Subcellular localization of IE1 N-terminal and C-terminal truncation mutants. pIZ-IE1_1__–__584_, pIZ-IE1_1__–__258_, pIZ-IE1_1__–__208_, pIZ-IE1_1__–__157_, pIZ-IE1_158__–__584_, or pIZ-IE1_209__–__584_ fused to express the HA tag was transfected into BmN-SWU1 cells. At 48 h p.t., immunofluorescence analysis was performed using an anti-HA antibody to observe the subcellular localization of the truncated mutants by a laser scanning confocal microscope. (**C**) Subcellular localization of EGFP fused with IE1 truncation mutants. IE1_1__–__584_, IE1_1__–__157_, IE1_158__–__208_, or IE1_209__–__584_ was fused with EGFP. pIZ-EGFP-IE1_1__–__584_, pIZ-EGFP-IE1_1__–__157_, pIZ-EGFP-IE1_158__–__208_, or pIZ-EGFP-IE1_209__–__584_ was transfected into BmN-SWU1 cells. At 48 h p.t., the subcellular localization of EGFP was observed by a laser scanning confocal microscope. (**D**) Subcellular localization of the intermediate segment deletion mutants IE1_1__–__157&209–__584_, IE1_1__–__157&259–__584_, IE1_1__–__157&309–__584_, IE1_1__–__157&369–__584_, IE1_1__–__157&429–__584_, IE1_1__–__157&479–__584_, IE1_1__–__157&539–__584_, or IE1_1__–__157&560–__584_ at 48 h p.t.

IE1, as the major transcriptional activator of baculovirus, is localized in the nucleus. A previous study reported residues 534–538 (basic domain II) as a nuclear localization signal of AcIE1 [14]. Here, the nuclear localization elements of BmNPV IE1 were analyzed. First, the overexpression vector pIZ-IE1_1–584_ and five truncated mutants, including pIZ-IE1_1–258_, pIZ-IE1_1–208_, pIZ-IE1_1–157_, pIZ-IE1_158–584_, and pIZ-IE1_209–584_, were fused to express the HA tag (Figure 3A). These vectors were transfected into BmN-SWU1 cells, and the subcellular localization of the different truncated mutants of IE1 was detected by immunofluorescence. The results showed that IE1_1–584_, IE1_1–258_, and IE1_1–208_ were completely localized in the nucleus, IE1_1–157_ was distributed in both the nucleus and cytoplasm, and IE1_158–584_ was completely localized in the nucleus. In contrast, IE1_209–584_ was localized in the nucleus and cytoplasm, suggesting that IE1_158–208_ functioned as a nuclear localization element (Figure 3B). Subsequently, IE1_1–584_, IE1_1–157_, IE1_158–208_, or IE1_209–584_ was fused with EGFP to construct the overexpression vectors pIZ-EGFP-IE1_1–584_, pIZ-EGFP-IE1_1–157_, pIZ-EGFP-IE1_158–208_, or pIZ-EGFP-IE1_209–584_, respectively, to verify whether they contained nuclear localization elements. The results of subcellular localization of EGFP revealed that EGFP fused with IE1_1–584_ or IE1_158–208_ was completely localized in the nucleus, whereas the EGFP fused with IE1_1–157_ or IE1_209–584_ was distributed in the nucleus and cytoplasm (Figure 3C), indicating that IE1_158–208_ was indeed a nuclear localization element of IE1, and that IE1_1–157_ and IE1_209–584_ contained minor nuclear localization elements. Next, a truncated mutant IE1_1–157&209––584_ lacking IE1_158–208_ was constructed to test whether other nuclear localization elements of BmNPV IE1 were sufficient to transport IE1 into the nucleus in addition to IE1_158–208_ (Figure 3A). It was found that IE1_1–157&209–584_ remained fully localized in the nucleus (Figure 3D), indicating that IE1_158–208_ was not an indispensable nuclear localization element of IE1. To identify other nuclear localization elements of BmNPV IE1, IE1_1–157&209–584_ was progressively truncated, and the truncated mutants were named IE1_1–157&259–584_, IE1_1–157&309–584_, IE1_1–157&369–584_, IE1_1–157&429–584_, IE1_1–157&479–584_, IE1_1–157&539–584_, and IE1_1–157&560–584_ (Figure 3A). The subcellular localization results revealed that IE1_1-157&259-584_, IE1_1-157&309-584_, IE1_1–157&369–584_, IE1_1–157&429–584_, IE1_1–157&479–584_, and IE1_1–157&539–584_ remained fully localized in the nucleus, while IE1_1–157&560–584_ was distributed in the nucleus and cytoplasm (Figure 3D), suggesting that IE1_539–559_ also contained a nuclear localization element. The above results indicate that IE1_158–208_ was a primary nuclear localization element, while IE1_1–157_ and IE1_539–559_ were minor nuclear localization elements.

### 2.4. Identification of Key Transactivation Domains of BmNPV IE1

**Figure 4:**
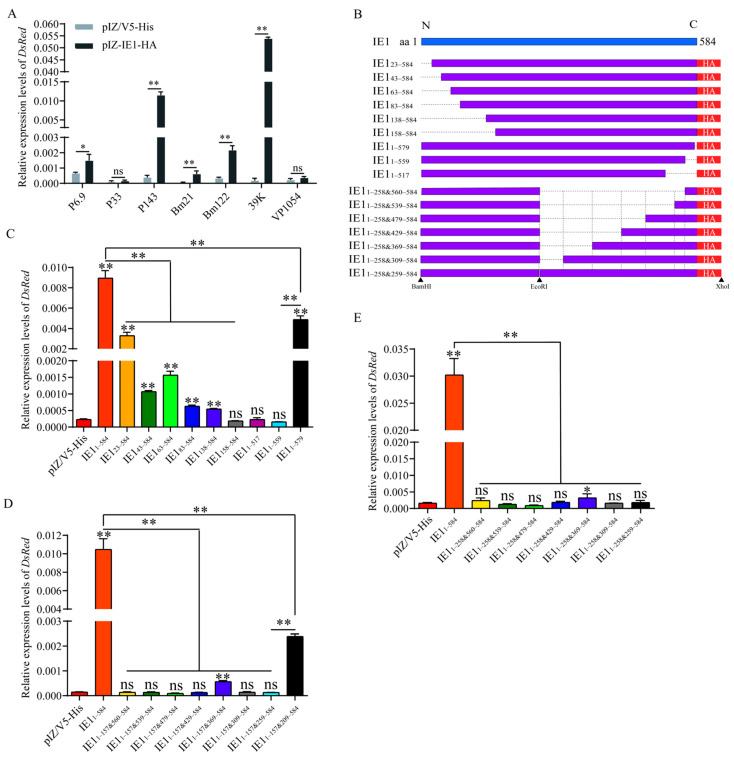
Identification of key functional domains for the transactivation of BmNPV IE1. (**A**) Transcriptional activation of different viral promoters by IE1. pIZ-IE1_1-584_ was co-transfected with pGL3-P6.9-DsRed, pGL3-P33-DsRed, pGL3-P143-DsRed, pGL3-Bm21-DsRed, pGL3-Bm122-DsRed, pGL3-39K-DsRed, or pGL3-vp1054-DsRed into BmN-SWU1 cells. At 48 h p.t., the transcriptional level of the reporter gene *DsRed* in each group was detected to reflect the transcriptional activation activity of IE1 (ns, no significance; * *p* < 0.05, ** *p* < 0.01). (**B**) Schematic of the construction of IE1 truncated mutants. (**C**) Transcriptional activation activity of IE1 N-terminal and C-terminal truncation mutants. pIZ-IE1_1__–__584_, pIZ-IE1_23__–__584_, pIZ-IE1_43__–__584_, pIZ-IE1_63__–__584_, pIZ-IE1_83__–__584_, pIZ-IE1_138__–__584_, pIZ-IE1_158__–__584_, pIZ-IE1_1__–__517_, pIZ-IE1_1__–__559_, or pIZ-IE1_1__–__579_ were co-transfected with pGL3-39K-DsRed into BmN-SWU1 cells. At 48 h p.t., the transcriptional level of *DsRed* by transactivation of IE1 truncation mutants was detected in each group (ns, no significance; ** *p* < 0.01). (**D**) Transcriptional activation activity of the intermediate segment deletion mutants containing IE1_1__–__157&560–__584_, or (**E**) intermediate segment deletion mutants containing IE1_1__–__258&560–__584_ at 48 h p.t. (ns, no significance; * *p* < 0.05, ** *p* < 0.01).

As the viral genes involved in viral DNA replication are early genes, these genes might not be synthesized to a sufficiently high concentration for viral DNA replication without transactivation activity of IE1. Therefore, the key transcriptional activation domains of BmNPV IE1 were further investigated. In our previous study, we demonstrated that BmNPV could induce the transcription of the viral gene promoters P6.9, P33, P143, Bm21, Bm122, 39K, and VP1054 [17]. As IE was a major transcriptional activator of baculovirus, we investigated whether IE1 has transcriptional activation for these promoters. The results of qRT-PCR demonstrated that BmNPV IE1 could induce transcription of the P6.9, P143, Bm122, and 39K promoters, except for P33 and VP1054 promoters, while IE1 had the highest transactivation activity on the 39K promoter (Figure 4A). Thus, the 39K promoter was used for the subsequent detection of IE1 transactivation activity. To identify the key functional domains of BmNPV IE1 with transactivation, nine truncated mutants of IE1_23-584_, IE1_43-584_, IE1_63-584_, IE1_83-584_, IE1_138-584_, IE1_158–584_, IE1_1–579_, IE1_1–559_, and IE1_1–517_ were constructed (Figure 4B). The overexpression vectors of IE1 truncated mutants were co-transfected with the pGL3-39K-DsRed reporter vector into BmN-SWU1 cells, and the relative expression levels of *DsRed* were measured by qRT-PCR at 72 h p.t. Consequently, with the progressive truncation of 157 amino acids at the N-terminus of IE1, the transcriptional levels of *DsRed* gradually decreased, indicating that the transcriptional activation of these truncated mutants gradually diminished, and the transactivation activity of IE1_158–584_ was completely lost, suggesting that the N-terminal 1–157 residues of IE1 were necessary for the transcriptional activation of IE1 (Figure 4C). Meanwhile, the transcript levels of *DsRed* were significantly lower in the IE1_1–579_ group than those in the IE1_1–584_ group, while the transcript levels of *DsRed* in the IE1_1–559_ and in the IE1_1–517_ groups were not remarkably different from those in the pIZ/V5-His group, highlighting that the 25 amino acids at the C-terminus of IE1 were also indispensable for the transactivation activity of IE1 (Figure 4C). We next detected the transactivation of the truncated mutant IE1_1–157&560–584_ to test whether IE1_1–157_ and IE1_560–584_ were fully competent for the transactivation activity of IE1 (Figure 4B). The result of qRT-PCR showed that IE1_1–157&560–584_ was insufficient to transactivate *DsRed* transcription, indicating that other elements are still required for the transactivation activity of IE1 (Figure 4D). To identify additional transactivating elements, the transactivation of the truncated mutants IE1_1–157&539–584_, IE1_1–157&479–584_, IE1_1–157&429–584_, IE1_1–157&369–584_, IE1_1–157&309–584_, IE1_1–157&259–584_, and IE1_1–157&209–584_ was detected. The transcript levels of *DsRed* in the IE1_1–157&539–584_, IE1_1–157&479–584_, IE1_1–157&429–584_, IE1_1–157&309–584_, and IE1_1–157&259–584_ groups were not significantly different from those in the pIZ/V5-His group, except for a limited increase in the IE1_1–157&369–584_ group (Figure 4D). The transcriptional level of *DsRed* in the IE1_1–157&209–584_ group was noticeably higher than that in the pIZ/V5-His and IE1_1–157&259–584_ groups, but only 22.82% of that in the IE1_1–584_ group, indicating that both IE1_209–258_ and IE1_158–208_ were important for the transcriptional activation activity of IE1, which was consistent with the complete loss of transactivation activity in the IE1_1–157&259–584_ group (Figure 4D). As IE1_158–258_ was required for the transactivation of IE1, a truncated mutant IE1_1–258&560–584_ was constructed to test whether it was sufficient for transactivation of IE1 (Figure 4B). The pIZ-IE1_1–258&560–584_ and pGL3-39k-DsRed vectors were co-transfected, and the relative expression of *DsRed* was detected at 72 h p.t. The results showed that in the IE1_1–258&560–584_ group, the relative expression of *DsRed* was similar to that in the pIZ/V5-His group, indicating that there are still other elements that are essential for the transactivation activity of IE1 (Figure 4E). Hence, the truncated mutants were further constructed by stepwise extension based on IE1_1–258&560–584_ (Figure 4B). However, all truncated mutants IE1_1–258&539–584_, IE1_1–258&479–584_, IE1_1–258&429–584_, IE1_1–258&309–584_, and IE1_1–258&259–584_ lost transcriptional activation ability, except for IE1_1–258&369–584_, which had a weak transcriptional activation (Figure 4E). These results suggest that the association of amino acids 258 and 259 is essential for the transcriptional activation activity of IE1. To examine whether the transactivation of IE1 was consistent with the effect of IE1 on viral proliferation, pIZ-IE1_1–584_ and the truncated mutants pIZ-IE1_23–584_, pIZ-IE1_43–584_, pIZ-IE1_63–584_, pIZ-IE1_1–559_, pIZ-IE1_1–579_, and pIZ-IE1_1–157&209–584_ were separately co-transfected with the *ie-1* deletion bacmid vBm^ie1-null^ to observe the repair effect of IE1 truncated mutants. The fluorescence observation showed that all truncated mutants except IE1_1–579_ could not promote the proliferation of vBm^ie1-null^ (Figure S3A). The results of viral DNA copy number detection by qRT-PCR were consistent with the results of fluorescence analysis (Figure S3B). The above results suggest that IE1_1–258_ and IE1_560–584_, and the association of amino acids 258 and 259 were essential for the transactivation activity of BmNPV IE1, and that IE1_1–22_ and IE1_158–208_ had other functions, which were required for BmNPV proliferation, besides acting as the functional domain of transcriptional activation.

## 3. Discussion

BmNPV and AcMNPV have approximately 93% amino acid sequence identity [18], but AcMNPV infects a significantly more diverse range of insects and insect cell lines than BmNPV; indeed, BmNPV is only highly infective to *Bombyx mori* and *Bombyx mori*-*derived* cell lines [19]. Baculoviruses are widely used in mammalian cells as gene delivery vectors, while a major barrier in the use of baculovirus vectors for therapeutic gene delivery is the viral inactivation by serum complement [20]. Meanwhile, IE1, a major transcriptional activator of baculovirus, is also functional in mammalian cells, and the loss of IE1 function could abrogate viral gene expression in baculovirus-transduced mammalian cells [21,22]. One study showed that the BmNPV vector was more stable in human serum than the AcMNPV vector in an complement inactivation assay in vitro [23]. Moreover, the study of BmNPV can better guide the antiviral research in the silkworm. Therefore, regardless of the similarity of AcMNPV and BmNPV, the studies of BmNPV and BmNPV IE1 are equally important.

IE1 is a major transcriptional activator, and BmNPV IE1 is also critical for BmNPV proliferation, which was investigated by constructing an IE1 deletion virus vBm^ie1-null^ and IE1 repair virus vBm^ie1-null-IE1(HA)^. However, the virulence of IE1 repair virus vBm^ie1-null-IE1(HA)^ did not reach the level of wild-type virus vBm^WT^. Another IE1 repair virus vBm^ie1-null-IE1prm2-IE1^ with higher promoter activity had higher virulence compared to vBm^ie1-null-IE1(HA)^, but was still lower than that of the wild-type virus vBm^WT^. The upstream and downstream genes of the BmNPV *ie-1* locus are *orf122* and *odv-e56*, respectively, where *orf122* is encoded by the negative strand of the BmNPV genome and its promoter is located in the coding region of *ie-1.* The construction of the *ie-1* deletion BmNPV bacmid was due to the substitution of *ie-1* 174–598 bp by the chloramphenicol resistance gene by homologous recombination, and this region was located 231–655 bp upstream of the *orf122* transcription start site. Indeed, deletion of the homolog of BmNPV *orf122* and AcMNPV *Ac146* eliminates the production of the budded virus [24]. The deletion of IE1 may impair the transcriptional activity of the *orf122* promoter, resulting in decreased expression of *orf122*, thereby affecting the virulence of IE1 repair virus vBm^ie1-null-IE1(HA)^ and vBm^ie1-null-IE1prm2-IE1^.

DNA replication of baculovirus takes place in the nucleus of infected host cells, which requires that many viral proteins must enter the nucleus in order to function. He et al. showed that 25 viral proteins were localized in the nucleus by transient transfection experiments in the absence of AcMNPV infection. Most of these proteins are involved in viral DNA replication, transcription, and virion structure, and 20 of them contain a predicted classical nuclear localization signal [25]. IE1 acts as an immediate early protein and major transcriptional activator, and nuclear localization signal is required for its function. Nuclear import elements of transcriptional activators often comprise basic residues [26]. AcIE1 contains two basic domains, basic domain I (residues 147–165) and basic domain II (521–542). It has been shown that the basic domain I is not essential for the nuclear localization of AcIE1, while the basic domain II was required for the nuclear localization of AcIE1 [14]. The N-terminus of BmNPV IE1_158–208_, as a primary nuclear localization element, contains a positively charged amino acid-rich motif KPKYKK, which is consistent with the basic domain I of AcIE1. Thus, although the basic domain I of AcIE1 is nonessential for the nuclear localization of AcIE1, whether the basic domain I of BmNPV IE1 is a nuclear localization signal needs to be further analyzed. Basic domain II residues R^537^ and R^538^ of AcIE1 are required for IE1 nuclear localization [14]. Moreover, BmNPV IE1_539–559_ as a secondary nuclear localization element includes the two arginines, which may explain why the absence of IE1_539–559_ weakened the nuclear localization of BmNPV IE1; however, these amino acids are not requisites for the nuclear import of BmNPV IE1. AcIE1 is predicted to have a bipartite nuclear localization signal at residues 130–151 [25], which may account for the ability of BmNPV IE1_1–157_ to partially enter the nucleus. Although we identified three nuclear localization elements of BmNPV IE1, further truncations and point mutations need to be performed to identify the key amino acids that perform nuclear localization.

Expression of baculovirus genes is a cascade-regulated process, and immediate early protein IE1 transactivation activity induces the transcription of many viral genes and determines the beginning of the viral life cycle. The transactivation of IE1 depends on the acidic activation domain and the DNA-binding domain. The acidic activation domain is located at the N-terminal end of IE1, and the DNA-binding domain is located at the C-terminal end of IE1 [8]. The deletion of 25 amino acids at the C-terminus of AcIE1 resulted in complete loss of DNA binding activity. The complete loss of transactivation activity of BmNPV IE1_1–559_ might be due to the deletion of IE1_560–584_ at its C-terminus, which disrupted the helix-coil-helix domain and abrogated the DNA binding ability [8,12]. In previous studies, residues 1–125 and residues 168–222 of AcIE1 were identified as independent transcriptional activation domains, while residues 152–161 were also required for AcIE1 transactivation, which is consistent with our results that BmNPV IE1_1–258_ was required for the transcriptional activation of BmNPV IE1 [9,10]. However, residues 169–266 of AcIE1 are dispensable for the transcriptional activity of residues 1–266 of AcIE1 [9], and our results showed that the deletion of BmNPV IE1_209–258_ led to a 94.9% decrease in the transcriptional activity of IE1_1–157&209–584_; thus, IE1_209–258_ is indispensable for the transcriptional activity of BmNPV IE1. The deletion of BmNPV IE1 1–23 residues decreased the transcriptional activity of IE1 by 63.1%, while AcIE1 1–23 residues were dispensable for transactivation [11]. Moreover, the deletion of IE1 1–23 residues had a more severe negative effect on BmNPV proliferation than on the transactivation of BmNPV IE1, perhaps because the phosphorylation of Thr^15^ is necessary for viral multiplication [11]. Unexpectedly, the transcriptional activity of BmNPV IE1_1–258&259–584_ was also lost, and whether residue 258 forms a key motif with residue 259 and their flanking amino acids is worthy of further investigation. Whether IE1 truncation mutant IE1_1–308&560–584_ are competent for the function of full-length IE1 and whether they can be further truncated is also an interesting topic.

Small molecule antagonists have important application prospects as therapeutic agents, and truncation of effector proteins by genetic engineering is an effective strategy [27]. A truncated mutant of a protein with a functional domain deletion can often act as an antagonist to disable the function of the protein. For instance, N- and C-terminally truncated forms of glucose-dependent insulinotropic polypeptide (GIP) are often used as competitive antagonists of the human GIP receptor [28]. N-terminally truncated glucagon fragments act as antagonists to decrease glucose in diabetic animals and patients [29]. The truncated adrenomedullin/adrenomedullin2 (ADM/ ADM2) analogs act as ADM/ADM2 antagonists to block CLR/RAMP signaling to prevent migraine pain and inhibit tumor growth/metastasis [30]. The truncated form of BmMPV IE1, which has nuclear localization signaling and DNA-binding ability such as IE1_158–208&560–584_ but lacks the transactivation domain, can theoretically enter the nucleus of BmNPV-infected cells normally and exerts competitive inhibition on IE1, thereby affecting BmNPV proliferation. BmNPV is a serious pathogen that infects silkworms, with no currently effective therapeutic available. A truncated form of IE1 small molecule deleting the transcriptional activation domain has the potential to be developed as a drug against BmNPV infection.

Inducible promoters are preferred over constitutive promoters because they are mostly reversible and more flexible for use in functional genomics, genetic engineering, and gene therapy [31]. In our previous studies, a baculovirus-inducible promoter, 39K, was screened and applied to establish a baculovirus-inducible Cas9 system and transgenic silkworms [32,33]. However, BmNPV infection causes cytopathic alteration or individual death in silkworm, which limits the application of the 39K promoter, except for silkworm antiviral research. IE1 protein is capable of activating 39K promoter transcription. The active small molecule BmNPV IE1 protein synthesized by binding to the key functional domains of IE1 may act as a 39K promoter agonist and constitute a foreign protein inducible expression system with the 39K promoter, which expands the application of the 39K promoter in the field of gene function and genetic engineering.

In summary, we analyzed the sequence features of BmNPV IE1 and determined that BmNPV IE1 is required for BmNPV proliferation. Additionally, BmNPV IE1_158–208_ was identified as a primary nuclear localization signal that fully mediated the entry of protein to the nucleus, and IE1_1-157_ and IE1_539-559_ were minor nuclear localization elements and partially mediated entry into the nucleus, while the combination of IE1_1–157_ and IE1_539–559_ was sufficient to transport protein into the nucleus. Meanwhile, we identified that BmNPV IE1_1–258_ and IE1_560–584_, and the association of amino acids 258 and 259 were necessary for the transcriptional activation activity of IE1. Although the sequence similarity between BmNPV IE1 and AcIE1 protein is as high as 95%, their functional domains show significant differences. Our study further elucidated the function of IE1 to provide a better understanding of baculovirus infection and new insights into the engineering and usage of IE1.

## 4. Materials and Methods

### 4.1. Sequence Characterization of BmNPV IE1

Protein sequence of BmNPV IE1 obtained from NCBI (GenBank accession numbers: NP_047544.1) was entered into the online software PSIPRED (http://bioinf.cs.ucl.ac.uk/psipred/, accessed on 12 April 2021) [34] for secondary structure prediction. The IE1 protein sequence then was entered into the online software SMART (http://smart.embl.de/, accessed on 12 April 2021) [35] to predict the domain of IE1. The protein sequences of IE1 derived from AcMNPV, opMNPV, CfMNPV, LdMNPV, and CpGV were obtained from NCBI with the GeneBank access numbers NP_054178.1, NP_046301.1, NP_848451.1, ANS70904.1, and NP_148791.1, respectively. These sequences were then put into a file and subsequently imported into ClustalX 2.1 for a complete alignment. Differences in amino acids indicated by different background colors represented the conservation of protein sequences. To construct a phylogenetic tree of BmNPV IE1, we used the BmNPV IE1 protein sequence for sequence alignment in NCBI to obtain the homologous sequence of BmNPV IE1 and imported them into MEGA-X 10.1.8 software for amino acid multiple sequence alignment and constructed the phylogenetic tree by Neighbor-Joining (NJ) method. The GeneBank access numbers of all IE1 proteins used were listed in Supplementary Table S1.

### 4.2. Cells and Transient Transfection

The *B. mori* ovary cell line, BmN-SWU1, was established and preserved at our laboratory [36], and was cultured at 27 °C with TC-100 medium (United States Biological, Swampscott, MA, USA) supplemented with 10% fetal bovine serum (BIOAGRIO, Mountain View, CA, USA), 100 U/mL penicillin, and 100 μg/mL streptomycin (Gibco, Grand Island, NY, USA).

BmN-SWU1 cells were plated in cell culture plates (Corning Incorporated, Corning, NY, USA) and grown to 80% confluence. Then, the plasmids or bacmids were transfected into the cells using TransIT^®^-Insect Transfection Reagent (Mirus, Madison, WI, USA) according to the manufacturer’s instructions.

### 4.3. Construction of ie-1 Deletion and ie-1 Repair Recombinant Viruses

The IE1 deletion bacmid was generated through homologous recombination in *E. coli* as previously described [37]. First, a transfer vector pSL1180-ie1US-Cm-ie1DS was constructed, in which the *ie-1* 174–598 bp was replaced by a chloramphenicol resistance gene (Cm) for antibiotic selection. Then, this transfer vector was digested, and the DNA fragment ie1US-Cm-ie1DS was transformed into *E. coli* BW25113 competent cells containing BmNPV bacmid, the recombinase vector pBAD-gbaA, and the transposase vector pMON7124 by heat stimulation. Next, the transformed cells were mixed with 800 μL of SOC medium and incubated at 37 °C for 6 h. The LB solid medium, including 50 μg/mL kanamycin, 7 μg/mL tetracycline, and 7 μg/mL chloramphenicol, was used to screen the positive colonies. Finally, monoclonal colonies were selected and the deletion of *ie-1* was detected by PCR using different primer pairs, including US-F/DS-R, ie1 ko-F/ie1 ko-R, Cm-F/Cm-R, ie1-US-F/Cm-R, and Cm-F/ie1-US-R.

Recombinant BmNPV with EGFP was constructed via the Bac-to-Bac system [38]. The plasmid pFastBac-Dual-P_PH_-polyhedrin-hsp-EGFP was previously constructed in our laboratory. First, the plasmid was transformed into DH10Bac Chemically Competent Cells (Weidi, Shanghai, China) or *E. coli* BW25113 Competent Cells containing IE1 deletion bacmid to construct recombinant virus vBm^WT^ or vBm^ie1-null^ with EGFP. For IE1 repair viruses, upstream sequences of *ie-1* CDS and *ie-1* CDS sequences were amplified from the viral genome using the primer pairs IE1prm-F/IE1-R or IE1prm2-F/IE1-R, and then ligated on the vector pFastBac-Dual-P_PH_-polyhedrin-hsp-EGFP to construct the vector pFastBac-Dual-P_PH_-polyhedrin-hsp-EGFP-IE1^prm^-IE1 or pFastBac-Dual-P_PH_-polyhedrin-hsp-EGFP-IE1^prm2^-IE1, which was then transformed into *E. coli* BW25113 Competent Cells containing IE1 deletion bacmid to construct the IE1 repair virus vBm^ie1-null-IE1(HA)^ or vBm^ie1-null-IE1prm2-IE1^ with EGFP. As mentioned earlier, the transformed cells were selected using LB solid medium supplemented with 50 μg/mL kanamycin, 7 μg/mL tetracycline, 7 μg/mL gentamicin, 40 μg/mL X-gal, and 40 μg/mL IPTG, and were validated by PCR using the primer pair M13F/M13R. All primers used to construct IE1 deletion and IE1 repair viruses are listed in Supplementary Table S2.

### 4.4. Plasmid Construction

To identify the nuclear localization elements of BmNPV IE1, a stepwise truncation was performed and all truncated fragments fused with HA tags were cloned into the insect expression vector pIZ/V5-His (Invitrogen, Carlsbad, CA, USA) to construct IE1 truncated mutants. The C-terminal truncated fragment included IE1_1–258_ (259–584 amino acids deletion), IE1_1–208_ (209–584 deletion), and IE1_1–157_ (158–584 deletion). The N-terminal truncated fragment included IE1_158–584_ (1–157 deletion) and IE1_209–584_ (1–208 deletion). The intermediate segment deletion included IE1_1–157&209–584_ (158–208 deletion), IE1_1–157&259–584_ (158–258 deletion), IE1_1–157&309–584_ (158–308 deletion), IE1_1–157&369–584_ (158–368 deletion), IE1_1–157&429–584_ (158–428 deletion), IE1_1–157&479–584_ (158–478 deletion), IE1_1–157&539–584_ (158–538 deletion), and IE1_1–157&560–584_ (158–568 deletion).

To identify the key functional domains of the transactivation of BmNPV IE1, a stepwise truncation analysis was performed and all truncated fragments fused with HA tags were cloned into pIZ/V5-His (Invitrogen) to construct IE1 truncated mutants. The N-terminal truncated fragment included IE1_23–584_ (1–22 amino acids deletion), IE1_43–584_ (1–42 deletion), and IE1_63–584_ (1–62 deletion), IE1_83–584_ (1–82 deletion), IE1_138–584_ (1–137 deletion), and IE1_158–584_ (1–157 deletion). The C-terminal truncated fragment included IE1_1–579_ (580–584 deletion), IE1_1–559_ (560–584 deletion), and IE1_1–517_ (518–584 deletion). The intermediate segment deletion included IE1_1–258&560–584_ (259–559 deletion), IE1_1–258&539–584_ (259–538 deletion), IE1_1–258&479–584_ (259–478 deletion), IE1_1–258&429–584_ (259–428 deletion), IE1_1–258&369–584_ (259–368 deletion), IE1_1–258&309–584_ (259–308 deletion), and IE1_1–258&259–584_ (0 deletions). All of the clones were verified by sequencing, and all of the primers that were used to construct IE1 truncated mutants are presented in Supplementary Table S3.

### 4.5. Immunofluorescence Assay

BmN-SWU1 cells were seeded on cover slips (Fisher Scientific, Waltham, MA, USA) in 24-well plates (Corning) and the plasmid was transfected into the cells by transfection reagent. At 48 h p.t., the cells were fixed with 4% paraformaldehyde for 15 min and were permeabilized in 0.1% Triton X-100 for 10 min, followed by washing three times with phosphate buffered saline (PBS) after each treatment. Then, the cells were blocked with PBS supplemented with 10% normal goat serum and 3% BSA for 1 h at 37 °C. Next, the cells were incubated with mouse anti-HA tag antibody (Beyotime, Shanghai, China) for 1 h at 37 °C and were incubated with Alexa Fluor 555-conjugated goat anti-mouse IgG and Hoechst 33342 (Life Technologies, Carlsbad, CA, USA) for 1 h at 37 °C, followed by washing six times with PBST after each treatment. Finally, the treated cells were imaged using a laser scanning confocal microscope (Olympus, Tokyo, Japan).

### 4.6. Western Blotting

BmN-SWU1 cells were transfected with the bacmids vBm^WT^, vBm^ie1-null^, and vBm^ie1-null-IE1(HA)^. At 24, 48, and 72 h p.t., the cells were lysed with western and IP cell lysis buffer (Beyotime), and the total proteins were harvested. After SDS-PAGE, the proteins were transferred to a polyvinylidene fluoride (PVDF) membrane (Millipore, Massachusetts, USA), blocked with TBST containing 10% skim milk powder, and then incubated with rabbit anti-VP39 or anti-tubulin antibody for 1 h (Beyotime). After washing six times with TBST, the blots were incubated with HRP-conjugated goat anti-rabbit IgG (Beyotime). After washing six times with TBST again, the Western blot results were analyzed with the an ECL Western Blotting Detection System (Bio-Rad, Hercules, CA, USA).

### 4.7. Real Time Fluorescent Quantitative PCR (qRT-PCR)

The cells transfected with plasmids or bacmids were harvested, and total RNA was prepared using the Total RNA Kit II (OMEGA, Norcross, GA, USA) and reverse transcribed into complementary DNA using PrimeScript™ RT reagent Kit with gDNA Eraser (Takara, Beijing, China). qRT-PCR was conducted in a 10 μL reaction mixture with NovoStart SYBR qPCR SuperMix Plus (Novoprotein, Shanghai, China), and each test was performed thrice. The reaction conditions were 95 °C for 30 s, followed by 40 cycles at 95 °C for 5 s and 60 °C for 30 s. Primers of the house-keeping gene, *silkworm translation initiation factor 4A* (sw22934), were used to normalize the gene expression. Sample analysis was performed on the qTOWER3G (Analytik Jena AG, Jena, Germany).

Total DNA was extracted using a Wizard Genomic DNA extraction kit (Promega, Madison, WI, USA). The *gp41* viral gene was used to quantify viral DNA abundance. qRT-PCR was performed as previously described. All primers used for qRT-PCR are listed in Supplementary Table S4.

### 4.8. Statistical Analysis

Statistical analysis was performed using GraphPad Prism 8 (GraphPad, San Diego, CA, USA). Student’s *t*-test was used to evaluate the statistically significant differences between different treatment groups. A *p*-value < 0.05 or *p* < 0.01 indicated a significant difference represented by “*” or “**”, respectively. Data are presented as the mean ± SD from at least three independent biological replicates.

## Data Availability

Not applicable.

## Supplementary Materials

The following supporting information can be downloaded at: https://www.mdpi.com/article/10.3390/ijms231810276/s1.
