# Supplementary material for: Identification of the Key Functional Domains of Bombyx mori Nucleopolyhedrovirus IE1 Protein
Source: Int J Mol Sci. 2022 Sep 7;23(18):10276. doi: 10.3390/ijms231810276 (PMC9499007; doi:10.3390/ijms231810276)
Supplement: Supplementary file 1 [file ijms-23-10276-s001.zip › ijms-1834896-supplementary.pdf]

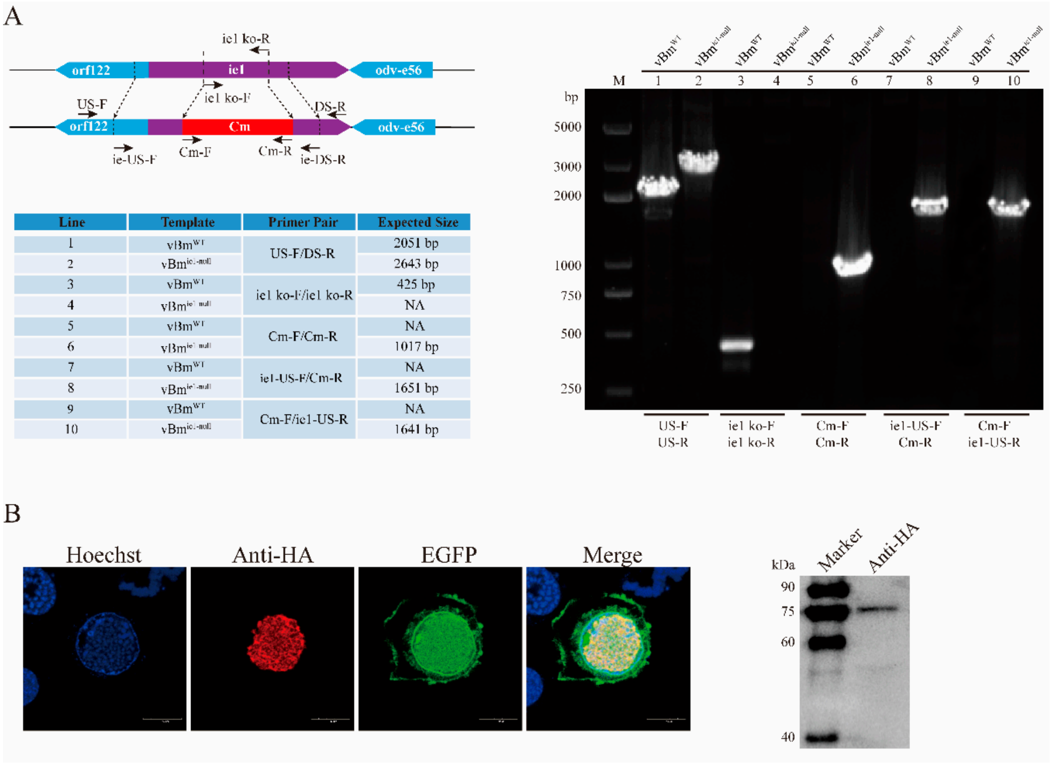

Line	Template	Primer Pair	Expected Size
1	vBm^{WT}	US-F/DS-R	2051 bp
2	$vBm^{ie1-null}$		2643 bp
3	vBm^{WT}	ie1 ko-F/ie1 ko-R	425 bp
4	$vBm^{ie1-null}$		NA
5	vBm^{WT}	Cm-F/Cm-R	NA
6	$vBm^{ie1-null}$		1017 bp
7	vBm^{WT}	ie1-US-F/Cm-R	NA
8	$vBm^{ie1-null}$		1651 bp
9	vBm^{WT}	Cm-F/ie1-US-R	NA
10	$vBm^{ie1-null}$		1641 bp

Supplementary Figure S1. Validation of the construction of IE1 deletion and IE1 repair viruses. **(A)** Validation of the construction of the IE1 deletion virus. Different primers were designed, as shown in the top left panel of Figure S1A, which were then combined, with the expected product sizes listed in the lower left panel of Figure S1A. Different DNA fragments were amplified using vBm^{WT} or $vBm^{ie1-null}$ bacmids as templates by the corresponding primer pair to determine whether the IE1 deletion virus was successfully constructed. (B) Validation of the construction of the IE1 repair virus. $vBm^{ie1-null-IE1(HA)}$ bacmids were transfected into BmN-SWU1 cells. At 72 h p.t., Immunofluorescence and western blotting were performed to verify the expression of IE1.

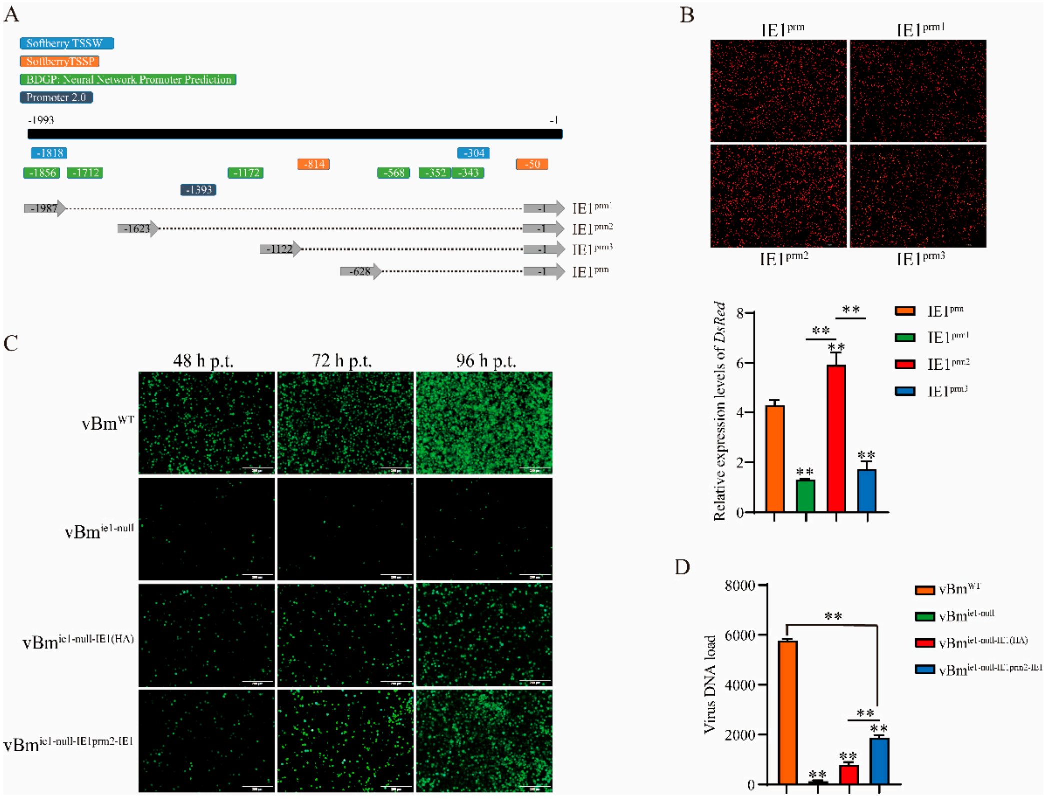

Supplementary Figure S2. Effect of promoter activity on the efficiency of IE1 repair. **(A)** Prediction of the IE1 promoter based on promoter prediction software. According to predictions from the websites Softberry TSSW, Softberry TSSP, and BDGP: Neural Network Promoter Prediction and Promoter 2.0, three promoters of different lengths were selected, namely, IE1^{prm1} (–1 to –1987), IE1^{prm2} (–1 to –1623), IE1^{prm3} (–1 to –1122). The promoter IE1^{prm} (–1 to –628) is the commercial IE1 promoter used in the previous section. **(B)** Promoter activity of different IE1 promoters. The promoter IE1^{prm1}, IE1^{prm2}, IE1^{prm3}, or IE1^{prm} was used to construct the reporter vector pSL1180-IE1^{prm1}-DsRed, pSL1180-IE1^{prm2}-DsRed, pSL1180-IE1^{prm3}-DsRed, or pSL1180-IE1^{prm}-DsRed, respectively. DsRed-positive cells were observed by fluorescence microscopy and the transcriptional levels of *DsRed* were examined at 48 h p.t. to determine the activity of the IE1 promoter (**$p < 0.01$). **(C)** Fluorescence observation of BmN-SWU1 cells. vBm^{WT}, vBm$^{ie1\text{-}null}$, vBm$^{ie1\text{-}null\text{-}IE1(HA)}$, and vBm$^{ie1\text{-}null\text{-}IE1prm2\text{-}IE1}$ bacmids were transfected into BmN-SWU1 cells, respectively. At 48, 72, and 96 h p.t., EGFP-positive cells were observed by fluorescence microscopy. **(D)** The virus DNA load in BmN-SWU1 cells transfected with vBm^{WT}, vBm$^{ie1\text{-}null}$, vBm$^{ie1\text{-}null\text{-}IE1(HA)}$, and vBm$^{ie1\text{-}null\text{-}IE1prm2\text{-}IE1}$ bacmids at 72 h p.t. (**$p < 0.01$).

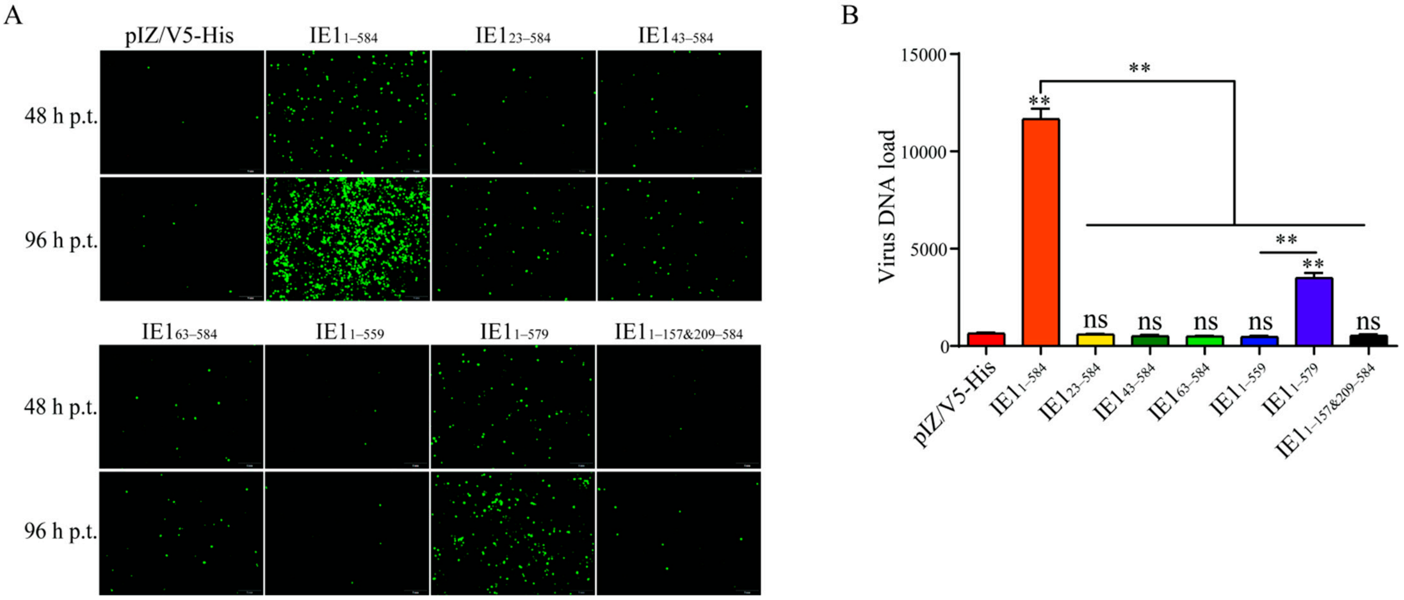

Supplementary Figure S3. Effect of IE1 truncation mutants on the proliferation of BmNPV. **(A)** Fluorescence observation of BmN-SWU1 cells. The plasmid pIZ-IE1$_{1\text{-}584}$, pIZ-IE1$_{23\text{–}584}$, pIZ-IE1$_{43\text{–}584}$, pIZ-IE1$_{63\text{–}584}$, pIZ-IE1$_{1\text{–}559}$, pIZ-IE1$_{1\text{–}579}$, or pIZ-IE1$_{1\text{–}157\&209\text{–}584}$ was co-transfected with the *ie-1* deletion bacmid vBm$^{ie1\text{-}null}$. At 48 and 96 h p.t., EGFP-positive cells were observed by fluorescence microscope to determine the proliferation of BmNPV. **(B)** The virus DNA load in BmN-SWU1 cells co-transfected with IE1 truncation mutants and vBm$^{ie1\text{-}null}$ bacmids at 96 h p.t. (ns, no significance; **$p < 0.01$).

Supplementary Table S1. The GeneBank Access Numbers of IE1 used in this study.

Species	Protein Description	GeneBank Access Number
Dendrolimus kikuchii nucleopolyhedrovirus	DekiNPV IE1	AFS51887.1
Thysanoplusia orichalcea nucleopolyhedrovirus	ThorNPV IE1	YP_007250550.1
Maruca vitrata nucleopolyhedrovirus	MaviNPV IE1	YP_950845.1
Bombyx mandarina nucleopolyhedrovirus	BomaNPV IE1	ACQ57320.1
Bombyx mori nucleopolyhedrovirus	BmNPV IE1	NP_047544.1
Rachiplusia ou multiple nucleopolyhedrovirus	RoMNPV IE1	AAN28024.1
Autographa californica nucleopolyhedrovirus	AcMNPV IE1	NP_054178.1
Antheraea yamamai nucleopolyhedrovirus	AnyaNPV IE1	BBD50465.1
Antheraea pernyi nucleopolyhedrovirus	AnpeNPV IE1	YP_610980.1
Epiphyas postvittana nucleopolyhedrovirus	EppoNPV IE1	NP_203298.1
Anticarsia gemmatalis multiple nucleopolyhedrovirus	AngeMNPV IE1	YP_009316031.1
Hyphantria cunea nucleopolyhedrovirus	HycuNPV IE1	YP_473198.1
Choristoneura murinana nucleopolyhedrovirus	ChmuNPV IE1	YP_008992101.1
Choristoneura rosaceana nucleopolyhedrovirus	ChroNPV IE1	YP_008378363.1
Spodoptera litura nucleopolyhedrovirus	SpliNPV IE1	NP_258284.1
Leucania separata nucleopolyhedrovirus	LeseNPV IE1	YP_758313.1
Spodoptera cosmioides nucleopolyhedrovirus	SpcoNPV IE1	QEI03567.1
Spodoptera litura nucleopolyhedrovirus II	SpliNPV IE1 II	YP_002332837.1
Spodoptera exigua multiple nucleopolyhedrovirus	SeMNPV IE1	AYN45080.1
Spodoptera frugiperda multiple nucleopolyhedrovirus	SfMNPV IE1	YP_001036428.1
Agrotis segetum nucleopolyhedrovirus B	AgseNPV IE1 B	YP_009112704.1
Agrotis ipsilon multiple nucleopolyhedrovirus	AgipMNPV IE1	YP_002268183.1
Agrotis segetum nucleopolyhedrovirus A	AgseNPV IE1 A	YP_529816.1
Mamestra brassicae multiple nucleopolyhedrovirus	MabrMNPV IE1	YP_009011216.1
Helicoverpa armigera multiple nucleopolyhedrovirus	HearMNPV IE1	ACH88677.1
Mamestra configurata nucleopolyhedrovirus A	MacoNPV IE1 A	QGX02407.1
Mythimna unipuncta nucleopolyhedrovirus	MyunNPV IE1	YP_009666803.1
Helicoverpa armigera nucleopolyhedrovirus	HearNPV IE1	ALD88580.1
Helicoverpa armigera nucleopolyhedrovirus G4	HearNPV IE1 G4	NP_075083.1
Helicoverpa zea single nucleopolyhedrovirus	HzSNPV IE1	AAB54100.1
Ectropis obliqua nucleopolyhedrovirus	EcobNPV IE1	YP_874199.1
Orgyia leucostigma nucleopolyhedrovirus	OrleNPV IE1	YP_001650917.1
Apocheima cinerarium nucleopolyhedrovirus	ApciNPV IE1	YP_006607883.1
Euproctis pseudoconspersa nucleopolyhedrovirus	EupsNPV IE1	YP_002854618.1
Adoxophyes orana nucleopolyhedrovirus	AdorNPV IE1	YP_002300536.1
Adoxophyes honmai nucleopolyhedrovirus	AdhoNPV IE1	NP_818666.1
Trichoplusia ni single nucleopolyhedrovirus	TrniSNPV IE1	AAL14888.2
Chrysodeixis chalcites nucleopolyhedrovirus	ChchNPV IE1	YP_249620.1
Lymantria dispar multiple nucleopolyhedrovirus	LdMNPV IE1	ANS70904.1

Lymantria xylina nucleopolyhedrovirus	LyxyNPV IE1	YP_003517752.1
Hemileuca sp. nucleopolyhedrovirus	HespNPV IE1	YP_008378231.1
Buzura suppressaria nucleopolyhedrovirus	BusuNPV IE1	YP_009001791.1
Clanis bilineata nucleopolyhedrovirus	ClbiNPV IE1	YP_717545.1
Diatraea saccharalis granulovirus	DisaGV IE1	YP_009182204.1
Adoxophyes orana granulovirus	AdorGV IE1	NP_872460.1
Cydia pomonella granulovirus	CypoGV IE1	NP_148791.1
Cryptophlebia leucotreta granulovirus	CrleGV IE1	NP_891854.1
Erinnyis ello granulovirus	ErelGV IE1	YP_009091847.1
Artogeia rapae granulovirus	ArraGV IE1	YP_003429330.1
Pieris rapae granulovirus	PiraGV IE1	AGS18772.1
Epinotia aporema granulovirus	EpapGV IE1	YP_006908543.1
Hyphantria cunea granulovirus	HycuGV IE1	QBQ01559.1
Agrotis segetum granulovirus	AgseGV IE1	YP_009513039.1
Spodoptera frugiperda granulovirus	SfGV IE1	YP_009121793.1
Mocis latipes granulovirus	MolaGV IE1	YP_009249846.1
Pseudalatia unipuncta granulovirus	PsunGV IE1	YP_003422347.1
Xestia c-nigrum granulovirus	XcGV IE1	NP_059157.1
Helicoverpa armigera granulovirus	HearGV IE1	YP_001648990.1

Supplementary Table S2. The primers used to construct IE1 deletion and IE1 repair viruses.

Primer name	Primer sequence
ie1-US-F (XhoI)	CCG***CTCGAG***GGACCTTGTGCTTTTGG
ie1-US-R (KpnI)	GG***GGTACC***GTCTCGCTGTCAGATACTA
ie1-DS-F (BamHI)	CG***GGATCC***CGCCGTATTTGATGCGTTTC
ie1-DS-R (EcoRI)	CG***GAATTC***GGGGATTGTCGGGAAACT
Cm-F (KpnI)	GG***GGTACC***TGTGTAGGCTGGAGCTGCT
Cm-R (BamHI)	CG***GGATCC***TCCATATGAATATCCTCC
US-F	AATCCTTGGCGTAGAA
DS-R	TCGCCAGAAATCCAATA
ie1-ko-F	TGCAGCAGCTTCAAACT
ie1-ko-R	CAAAATCATTAGTAAAA
IE1prm-F (BamHI)	CG***GGATCC***TTGCAGTTCGGGAC
IE1prm2-F (BamHI)	CG***GGATCC***AACAAAGCAGACACTCGGCA
IE1-R (SpeI)	GG***ACTAGT***TTA**AGCGTAATCTGGAACATCGTATGGG-TA**ATTAAATTCAATTTTTTTATATTTA
M13-F	CGCCAGGGTTTTCCCAGTCACGAC
M13-R	CAGGAAACAGCTATGACC

The sequences in italics and bold were restriction enzyme sites, bold sequences were HA tag sequences.

Supplementary Table S3. The primers used to construct IE1 truncated mutants.

Primer name	Primer sequence
IE1-F (BamHI)	CG***GGATCC***ATGACGCAAATTAATTTTAACG
IE1-R (XhoI)	CCG***CTCGAG***ATTAAATTCAATTTTTTTATATTTACAAT
HA-F (XhoI)	CCG***CTCGAG*****TACCCATACGATGTTCCAGATTACGCT**TAAT
HA-R (XbaI)	GC***TCTAGA***TTA**AGCGTAATCTGGAACATCGTATGGGTA**C
IE1-258R (EcoRI)	CG***GAATTC***GTAATAGTTGTTTGT
IE1-208R (EcoRI)	CG***GAATTC***GTCGTCGAAACGCATC
IE1-157R (EcoRI)	CG***GAATTC***CAATTTATTCTT
IE1-158F (BamHI)	CG***GGATCC***ATGAAGCCTAAATACAAGAAAAG
IE1-209F (BamHI)	CG*GGATCC*ATGAACGACTACAATTCCAACAGGT
IE1-209F (EcoRI)	CG***GAATTC***ATGAACGACTACAATTCCAACAGGT
IE1-259F (EcoRI)	CG***GAATTC***ATGGTAGATAATCGTGT
IE1-309F (EcoRI)	CG***GAATTC***ATGTTTGTCGATGTGCAT
IE1-369F (EcoRI)	CG***GAATTC***ATGCTTAGTCGTAAAGAGAGT
IE1-429F (EcoRI)	CG***GAATTC***ATGAAATACAGTAGTG
IE1-479F (EcoRI)	CG***GAATTC***ATGAATGTAAAAGGTCAC
IE1-539F (EcoRI)	CG***GAATTC***ATGCGAAGAGAGAGCACT
IE1-560F (EcoRI)	CG***GAATTC***CTGGTTCCGTTGTCC
IE1-23F (BamHI)	CG***GGATCC***ATGAACGGCTATTCAGAGT
IE1-43F (BamHI)	CG***GGATCC***ATGAATCCCACGCCGG
IE1-63F (BamHI)	CG***GGATCC***ATGAACTTTTTGGCAAGCGTCA
IE1-83F (BamHI)	CG***GGATCC***ATGAAGACCACTGATAATCTCGGA
IE1-138F (BamHI)	CG***GGATCC***ATGCTGGACGAATACTTGGAC
IE1-158F (BamHI)	CG***GGATCC***ATGAAGCCTAAATACAAGAAAAG
IE1-579R (XhoI)	CC***GCTCGA***GTTTATATTTACAA
IE1-559R (XhoI)	CC***GCTCGA***GACCCTGTAATATTAAAGCTA
IE1-517R (XhoI)	CC***GCTCGA***GAGCGTCTACATCTTT

The sequences in italics and bold were restriction enzyme sites, bold sequences were HA tag sequences.

Supplementary Table S4. The primers used for qRT-PCR.

Primer name	Primer sequence
RT-me53-F	CACAAAGAGCCCAACGAGC
RT-me53-R	GCCGCCGTCCAATACCTC
RT-gp64-F	CACCATCGTGGAGACGGACTAC
RT-gp64-R	ACCTCGCACTGCTGCCTGA
RT-vp39 -F	AGACACCACAAACCCGAACAC
RT-vp39 -R	TTGATCGCCAACACCACCT
RT-p10-F	AGACGCCATTGCGGAAAC
RT-p10-R	CGGGCAAACCGTCCAAAG
RT-gp41-F	ATGTTGATGTGCGGAAAGC
RT-gp41-R	GTGGCGGAATCGGTGA
RT-DsRed-F	GAGACCCACAAGGCCCTGA
RT-DsRed-R	CGGTGCGCTCGTACTGCT
RT-SW22934-F	TTCGTACTGGCTCTTCTCGT
RT-SW22934-R	CAAAGTTGATAGCAATTCCCT
